# Evaluation of the thickness of the proximal femoral canal in patients living at altitude

**DOI:** 10.1590/1413-78522015230200936

**Published:** 2015

**Authors:** Wiliam Soltau Dani, Marcos Emilio Kuschnaroff Contreras, Eleazar Lara Padilha, Francisco José Berral

**Affiliations:** 1 Clinitrauma, Lages, SC, Brazil, 1. Clinitrauma, Lages, SC, Brazil; 2 Hospital Governador Celso Ramos, Orthopedics Service, Florianópolis, SC, Brazil, 2. Orthopedics Service, Hospital Governador Celso Ramos, Florianópolis, SC, Brazil; 3 Instituto Politécnico Nacional, Mexico City, Mexico, 3. Instituto Politécnico Nacional, Mexico City, Mexico; 4 Universidad Pablo de Olavide, Seville, Spain, 4. Universidad Pablo de Olavide, Seville, Spain

**Keywords:** Altitude, Hip, Arthroplasty

## Abstract

**OBJECTIVE:**

Our goal is to confirm the hypothesis that people who were born and raised on cities at altitude have a smaller proximal femoral canal.

**METHODS:**

Prospective study with 169 participants, divided into two groups. Group A: 99 patients who were born and raised at altitude and group B: 70 patients who were born and raised at low altitude. All patients underwent panoramic radiographs of the pelvis, where we marked three measure and checked the thickness of the cortical and the lateral and medial cortical, as well as the thickness of the femoral canal.

**RESULTS:**

We noticed that the first measure showed no significant difference in both groups, but the second measure, the lateral cortex, is thicker in group A, and the femoral canal is smaller in comparison to group B.

**CONCLUSION:**

We concluded that patients who were born and raised at altitude have a smaller femoral canal. This may help in proper planning of future surgical procedures, especially in total hip arthroplasty cases.

**Level of Evidence II, Development of Diagnostic Criteria in Consecutive Patients (with universally applied reference "gold" standard).:**

## INTRODUCTION

Several studies are currently published relating metabolic and systemic changes in patients living at high altitudes, mainly related to hipoxia.^1^^-^ 4

The genotypic and phenotypic relationship for these changes is well documented, especially in the Andean region and the Tibetan plateau.^5^^-^ 10

Studies show the difference in sport performance in athletes at altitude,^11^ as well as changes in the musculature of these pacientes.^12^^-^ 14

Comparisons with healthy people living at sea level, climbers and athletes to determine the effects of high altitude are reported, but there is no correlation with the bone structure described.

Because of increased life expectancy and the increasingly frequent appearance of degenerative processes of the joints, including the hips, and the need for arthroplasty replacement of these joints, we noticed that people who were born and raised at altitude have a smaller femoral canal as compared to other regions at low altitude or sea level.

Our goal is to confirm the hypothesis that the size of the proximal femoral canal is lower in this population. 

## MATERIALS AND METHODS

This is a prospective study of individuals living at altitude regions, at sea level or low-altitude regions of both genders aged between 20 and 60 years old without systemic bone diseases or at the hip level. The study was approved by the Institutional Ethics Committee and the patients signed a Free and Informed Consent Term.

We considered altitude cities those located at more than 800 meters above sea level and low altitude cities those located at maximum 150 meters in relation to sea level.

We divided the sample into two groups: Group A: 99 patients who were born and raised at altitude regions Group B: 70 patients who were born and raised at sea level or at low altitudes.

We applied a questionnaire investigating gender, age, dominant hand, race, ancestry, hometown, city where the individual was raised up to 21 years old and questions on the exclusion criteria.

We excluded from the study cases without full documentation (data and image tests), patients who did not fit into the age range, who had congenital disease in the proximal femur, high body mass index (BMI), rheumatic diseases, previous fracture at the proximal femur bone, metabolic disorders or that have undergone any prior procedure on the hip. All patients also had an adequate calcium intake and were engaged in regular physical activity.

All patients underwent panoramic digital radiographs of the pelvis using the following criteria: patient in supine position, radius of about 70 cm high, directed to the midline, just above the pubic symphysis, with feet turned inward about 15 to 20 degrees, the middle portion of the sacrococcygeal junction located in the same vertical axis of the symphysis, 2 to 5 cm distally from it; the trochanters not being superimposed to the femoral necks and the smaller trochanter being visible, but not protruding.

We evaluated the radiographs of the pelvis of the two groups with the following measures:

First measure: to the small trochanter level (measure of the lateral cortex, medial cortical and size of the femoral canal).

Second measure: 5 cm below the lesser trochanter (measure of the lateral cortex, medial cortical and size of the femoral canal).

Third measure: 10 cm below the lesser trochanter (measure of the lateral cortex, medial cortical and size of the femoral canal).

**Figure 1.**
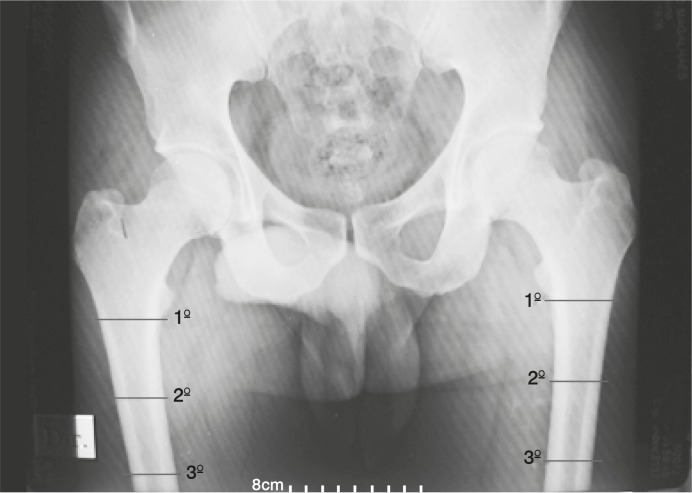
X-Ray showing the locations of the measurements performed.

All measurements were evaluated by the same observers. (Figure 1)

Statistical Analysis

We used the Kolmogorov-Smirnov normality test, with the PROC UNIVARIATE NORMAL SAS software (SAS Institute, 1999) for statistical evaluation of continuous variables. The comparison between means of the populations at different altitudes was performed using the Student t-test.

The decision on t-tests was determined from the variance homogeneity test (F test). We used the exact t-test when the variances were homogeneous. In non-homogeneous cases, we adopted the approximate t-test.

## RESULTS

We evaluated 169 patients, of these, 99 patients (58.58%) belong to municipalities at altitudes and 70 patients (41.42%) to municipalities at the sea level.

**Figure 2.**
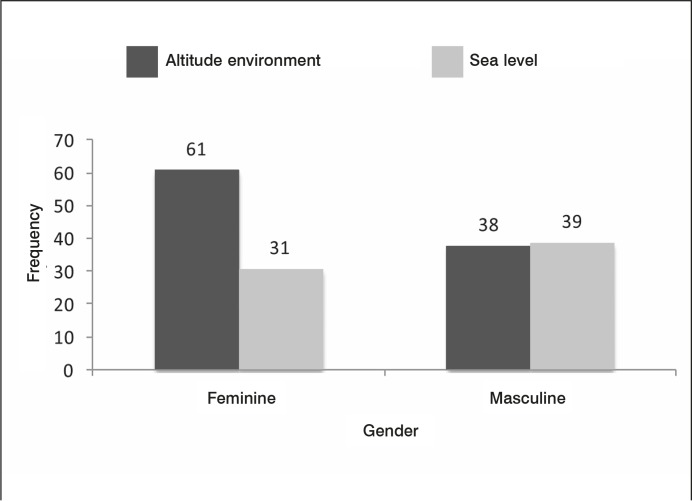
Frequency of patients evaluated in municipalities situated at altitude environment (n=99) and at sea level (n=70).

Of the 99 patients in municipalities at altitude, 61.62% (61 patients) were female and 38.38% (38 patients) were male. Of the 70 cases at sea level 44.3% (31 patients) were female and 55.7% (39 patients) were male. There was no statistically significant difference between genders comparing the femoral canal, but in male patients, the lateral and medial cortical has always been thicker. (Figure 2)

Patients at altitudes were aged between 20 and 60 years old. Patients of municipalities located at sea level were aged 22 to 60 years old. For both environments (altitude and sea level), more than 70% of patients were between 30 and 59 years old, with approximately 30% of them belonging to the age group 30-39 years old.

Right handed were 95.27% (n=161) of the sample, whereas patients with left dominant side contributed with only 4.73% (n=8) of the sample, and there was no statistically significant difference in the thickness of the femoral canal regarding the dominant side.

**Figure 3.**
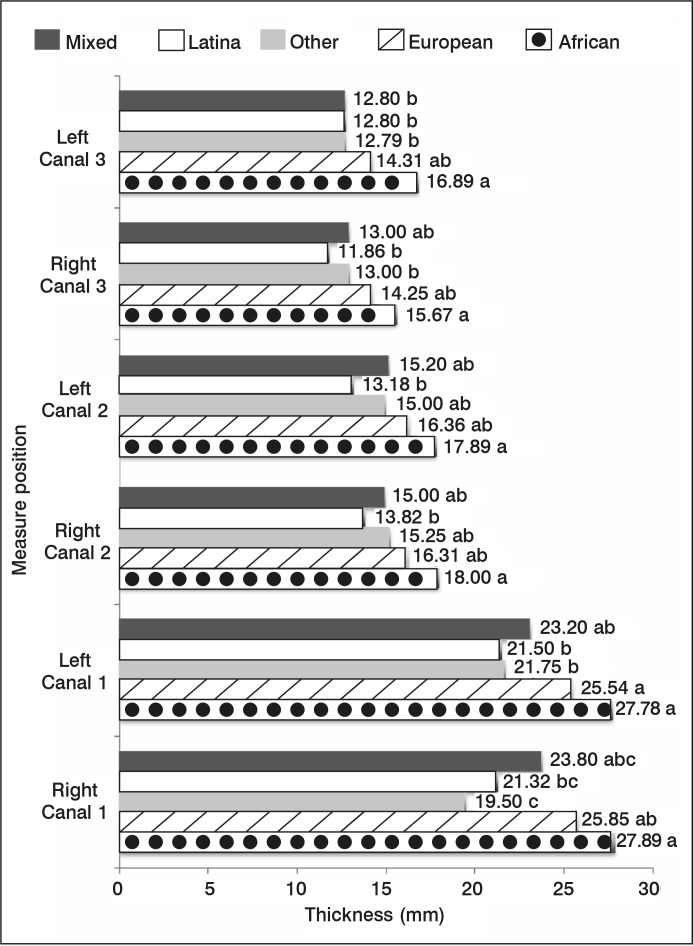
Mean thickness of femur canal in patients from different ancestries. Mean followed by the same letter do not differ according to Bonferroni test (p

As the contribution of ancestries to the sample, we observed that European, Latin, African, mixed descent and others corresponded to 129 (76.33%), 22 (13.01%), 9 (5.33%), 5 (2.96%) and 4 (2.37%) of patients, respectively. Comparing the population ancestries, we noticed that patients of African origin have significantly thicker femoral canal than patients from other origins. This behavior of the African group occurred in both groups. (Figure 3) 

The average altitude above the sea in group A was 948.32 meters and in group B of 34.26 meters.

**Figure 4.**
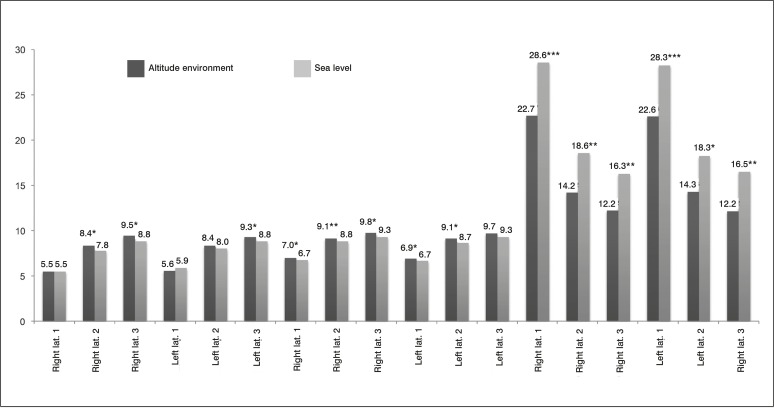
Estimated means (± Standard deviation) for the variables assessed in patients from cities at altitude and at sea level

Evaluating Figure 4 and Table 1 we noticed that in the first measurement there was no statistically significant variation in both groups. In the second and third measurements, however, there is a thickening of the lateral cortex in patients from the altitude group (group 1) and consequent thinning of the femoral canal in group 1 as compared to group 2.

**Table 1.** Estimated means (± standard deviation) for the variables assessed in patients from cities at altitude and at sea level, according to gender and place.

Variable	Altitude			Sea level		
	F	M	p	F	M	p
Age	46.61±12.41	41.58±11.64	0.005	37.55±12.06	43.80±11.60	0.03
Right Lat.1	5.31±0.87	5.86±1.07	0.005	5.35±0.98	5.61±1.21	0.33
RightMed.1	6.79±1.20	7.26±1.27	0.05	6.35±1.08	7.05±1.15	0.01
Right Can. 1	22.26±3.12	23.42±4.58	0.18	27.52±2.68	29.51±2.51	0.002
Left Lat.1	5.38±0.87	5.88±1.10	0.01	6.09±3.81	5.67±1.15	0.55
Left Med. 1	6.73±1.14	7.21±1.19	0.05	6.35±0.91	6.92±0.93	0.01
Left Can. 1	22.23±2.78	23.24±3.02	0.09	26.97±2.74	29.39±2.48	0.0002
Right Lat.2	8.00±1.30	8.92±1.71	0.003	7.45±1.21	8.03±1.37	0.07
Right Med.2	8.78±1.60	9.63±1.40	0.001	8.26±0.93	9.21±1.28	0.001
Right Can. 2	14.16±1.81	14.29±1.87	0.74	17.65±2.06	19.29±2.14	0.002
Left Lat.2	8.07±1.54	8.87±1.54	0.008	7.74±1.26	8.21±1.30	0.14
Left Med.2	8.75±1.54	9.66±1.49	0.005	8.42±0.92	8.78±1.06	0.05
Left Can. 2	14.23±2.06	14.47±1.93	0.56	17.52±2.08	18.87±3.57	0.05
Right Lat.3	9.15±1.39	9.95±1.65	0.01	8.41±1.39	9.13±1.30	0.03
Right Med.3	9.52±1.50	10.21±1.46	0.03	8.97±1.33	9.64±1.50	0.05
Right Can.3	12.08±1.48	12.47±1.74	0.26	15.42±1.82	17.23±2.20	0.001
Left Lat.3	9.02±1.33	9.79±1.55	0.01	8.42±1.41	9.18±1.27	0.02
Left Med.3	9.46±1.42	10.21±1.61	0.02	9.19±1.10	9.38±1.51	0.56
Left Can.3	12.08±1.64	12.40±1.81	0.38	15.42±1.93	17.41±2.79	0.001

## DISCUSSION

Degenerative diseases are more common in our midst due to increased life expectancy. Every day several surgeries are performed for replacement of joints with osteoarthritis through arthroplasty, especially in the hip.

During the process of preoperative planning for performing arthroplasty, we observed that patients that grew up at altitude had a reduced proximal femoral canal size, often causing technical difficulty to perform the procedure.

In reviewing the literature, we found no study showing changes in the femoral canal, and very few on bone structure with increasing altitude. All studies published refer to metabolic or genetic changes at high altitude.

Edwards *et al*.^1^ and Holm *et al*.,^13^ in their works, showed that in response to hypoxia at high altitudes, there is muscle atrophy, but there is no loss of function.

Wehby *et al*.^3^ comparing children's weight at birth at altitude intervals from 5 to 1280 meters and 1854 to 3600 meters, noticed that in the second interval the children had a lower weight after birth.

Several studies^5^^-^ 10 indicate genotypic and phenotypic changes in people living at altitude, especially in the Andean and Tibetan plateaus.

McSharry^11^ compared the performance of professional soccer players and the influence on game results at high altitudes, concluding that altitude provides a significant advantage to the teams that reside there.

Many works^15^^-^ 17 prove that there is a reduction in the ability to perform exercise at high altitude compared to the same activities at sea level.

In our study there was no difference in the thickness of the femoral canal comparing gender and dominant side in both groups. Regarding ancestry, the African group showed a significantly greater thickness of the femoral canal than patients from other origins. This behavior of the African group occurred in both groups. In the other ancestries, there was no statistically significant variation between the two groups.

Comparing the measurements of cortical and femoral canal from the first measurement to the third, we noticed that in measure 1 there was no statistically significant variation between the two groups.

From the second measure on, there was a thickening of the lateral cortex (measures 2 and 3) at the altitude group, as well as a decrease in the femoral canal. This fact makes us to consider that the accountability for the reduced femoral canal at altitude was a thickening of the lateral cortex of the proximal femur in the patients studied.

## CONCLUSION

Patients who were born and raised at altitude have a smaller femoral canal, due to a thickening of the lateral cortex. This might help in proper planning for future surgical procedures, in particular the cases of total hip arthroplasty.
